# Inflammatory Exophthalmos

**Published:** 1890-05-03

**Authors:** 


### INFLAMMATORY EXOPHTHALMOS.

It will not be uninteresting to compare with this remarkable case another that occurred soon afterwards in the practice of Dr. Eissen, in which the exciting causes were similar but the effects far less serious. A school-girl, aged 13, while at work in the harvest-field on a very hot day, was seized with acute pain in both eyes. She had drunk rather too freely of beer, which, with the exposure to the sun, brought on next day a violent attack of vomiting. The pain in the eyes was much aggravated thereby, and continued to increase until the sixth day, when she was brought to Dr. Eissen. The right eyeball had been pushed prominently forwards and outwards, and was quite immovable; the upper lid, œdematous and closely applied to the ball, could not be raised. The conjunctivæ were but moderately injected, the cornea was clear, and the iris was sensitive to light. The retina was somewhat clouded, and its vessels could be seen pulsating strongly. She was ordered rest in bed, with bladders of ice over the eyes. The pain soon abated, a sensation of pricking only remaining for some days, the exophthalmos rapidly subsided, and she was discharged cured in three weeks.

